# Effects of IGF-1 on Proliferation, Angiogenesis, Tumor Stem Cell Populations and Activation of AKT and Hedgehog Pathways in Oral Squamous Cell Carcinoma

**DOI:** 10.3390/ijms21186487

**Published:** 2020-09-05

**Authors:** Jéssica Mariane Ferreira Mendes, Ludmila de Faro Valverde, Manuela Torres Andion Vidal, Bruno Diaz Paredes, Paulo Coelho, Kyan James Allahdadi, Ricardo Della Coletta, Bruno Solano de Freitas Souza, Clarissa Araújo Gurgel Rocha

**Affiliations:** 1 Gonçalo Moniz Institute, Oswaldo Cruz Foundation (FIOCRUZ), Salvador, Bahia 40296-710, Brazil; jmarianemendes@gmail.com (J.M.F.M.); luludfaro@gmail.com (L.d.F.V.); manuela.andion@gmail.com (M.T.A.V.); paulocoelhoba@gmail.com (P.C.); 2 Center for Biotechnology and Cell Therapy, São Rafael Hospital, Salvador, Bahia 41253-190, Brazil; brunoparedes@gmail.com (B.D.P.); kyan.allahdadi@gmail.com (K.J.A.); 3 D’Or Institute for Research and Education (IDOR), Rio de Janeiro 22281-100, Brazil; 4 Department of Oral Diagnosis, School of Dentistry, Campinas State University (UNICAMP), Piracicaba, São Paulo 13414-903, Brazil; coletta@unicamp.br; 5 Department of Pathology, School of Medicine and School of Dentistry, Federal University of Bahia (UFBA), Salvador, Bahia 40110-909, Brazil

**Keywords:** IGF-1, hedgehog, PI3K-AKT

## Abstract

(1) Background: Activation of the PI3K-AKT pathway controls most hallmarks of cancer, and the hedgehog (HH) pathway has been associated with oral squamous cell carcinoma (OSCC) development and progression. We hypothesized that fibroblast-derived insulin-like growth factor-1 (IGF-1) acts in oral squamous cell carcinoma (OSCC) cells, leading to the non-canonical activation of the HH pathway, maintaining AKT activity and promoting tumor aggressiveness. (2) Methods: Primary fibroblasts (MF1) were genetically engineered for IGF-1 overexpression (MF1-IGF1) and CRISPR/Cas9-mediated IGF1R silencing was performed in SCC-4 cells. SCC-4 cells were co-cultured with fibroblasts or incubated with fibroblast conditioned medium (CM) or rIGF-1 for functional assays and the evaluation of AKT and HH pathways. (3) Results: Gene expression analysis confirmed IGF-1 overexpression in MF1-IGF1 and the absence of IGF-1 expression in SCC-4, while elevated IGF1R expression was detected. IGF1R silencing was associated with decreased survival of SCC-4 cells. Ihh was expressed in both MF1 and MF1-IGF1, and increased levels of GLI1 mRNA were observed in SCC-4 after stimulation with CM-MF1. Activation of both PI3K-AKT and the HH pathway (GLI1, Ihh and SMO) were identified in SCC-4 cells cultured in the presence of MF1-IGF1-CM. rIGF-1 promoted tumor cell proliferation, migration, invasion and tumorsphere formation, whereas CM-MF1 significantly stimulated angiogenesis. (4) Conclusions: IGF-1 exerts pro-tumorigenic effects by stimulating SCC-4 cell proliferation, migration, invasion and stemness. AKT and HH pathways were activated by IGF-1 in SCC-4, reinforcing its influence on the regulation of these signaling pathways.

## 1. Introduction

Oral squamous cell carcinoma (OSCC) is a growing global health issue due to increasing incidence, high mortality, limited therapeutic options and heterogeneous clinical responses to currently available treatments [1,2]. Therefore, the investigation of molecular pathways associated with tumor aggressiveness and the identification of novel therapeutic targets have become high priorities.

Insulin-like growth factor 1 (IGF-1) is a peptide that binds to a tyrosine kinase receptor (IGF1R) and promotes the phosphorylation of intracellular substrates, including PI3KCA, AKT-PKB, RAS/RAF/MAPK and p70S6 kinase, which triggers the nuclear translocation of transcription factors that regulate cell growth, differentiation and apoptosis, e.g., FOXO, GSK3β, MDM2 and mTOR [3,4,5]. In the tumor microenvironment, IGF-1 drives migration, invasion and proliferation [6,7], promotes angiogenesis and maintains cancer stemness [8]. The latter is regulated by direct or indirect mechanisms and by interaction with other embryonic signaling pathways, such as the hedgehog pathway [9].

To identify frequently altered genes in OSCC related to signaling pathways, we analyzed TCGA data from OSCC samples (available at CbioPortal) [10,11]. The amplification of genes encoding PIK3CA and PRKCI was observed in 15% of these tumors, suggesting the activation of the PI3K pathway, one of the most recognized sources of human tumor pathogenesis [12]. Data have also revealed that PI3K activation can regulate Glioma-associated oncogene 1 (GLI1) via hedgehog pathway (HH) transcription factor (Figure S1), which is directly related to OSCC development and progression [13,14]. This signaling cascade has been reported to be initiated by IGF-1 [3,4]

In tumoral stroma, IGF-1 is mainly secreted by cancer-associated fibroblasts (CAFs). These cells establish a favorable environment for tumor development by releasing cytokines (IL-6, IL-10 and TGF-β), metalloproteinases (MMP3 and MMP9) and growth factors (HGF, EGF, CTGF and IGF-1), leading to immunomodulation, angiogenesis, invasion, proliferation and tumor cell survival [15,16,17]. Considering that CAFs promote a pro-tumorigenic microenvironment through the secretion of several molecules, including IGF-1, the present study aimed to investigate the role of IGF-1 in cell proliferation, migration, angiogenesis, invasion and cancer cell stemness in OSCC, and to link these events to IGF-1-mediated regulation of AKT and HH pathways. Elucidating the mechanisms mediated by IGF-1 in the AKT and HH signaling pathways will contribute to the consolidation of IGF-AKT-HH blockade as a potential therapeutic target for OSCC.

## 2. Results

**Figure 1:**
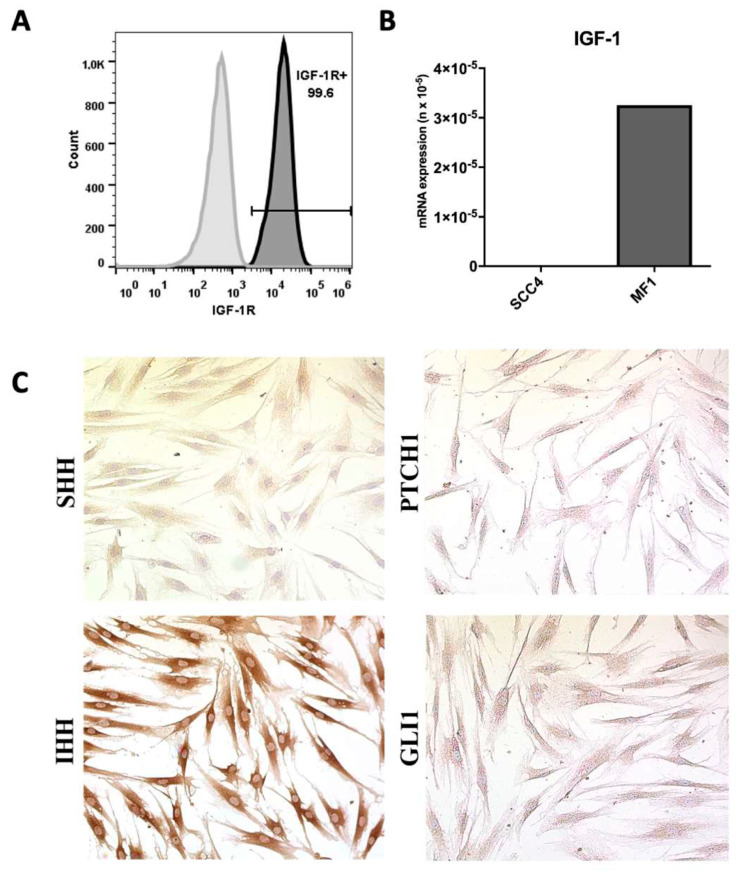
Evaluation of IGF-1, IGF1R and HH pathway expression in fibroblasts and oral squamous carcinoma cells. (**A**) Representative histogram of IGF1R (CD221, dark histogram) expression in SCC-4 cells assessed by flow cytometry. Unstained cells were used as controls (lighter histogram). (**B**) IGF1 mRNA expression in SCC-4 and MF1 cells, evaluated by RT-qPCR. (**C**) Evaluation of HH pathway component expression in MF1 by immunocytochemistry. Magnification: 400×.

In vitro studies were performed to evaluate the status of the IGF-1 signaling pathway in OSSC cells. First, IGF1R expression was evaluated in the SCC-4 OSCC cell line by flow cytometry. Histogram analysis demonstrated that 99.6% of tumor cells presented high IGF1R expression (Figure 1A). In order to evaluate whether IGF-1 is produced by tumor and stromal cells, RT-qPCR analysis was performed in SCC-4 and primary human fibroblasts (MF1). While MF1 expressed high amounts of IGF-1 mRNA, no amplification was detected in SCC-4 cells (Figure 1B). In addition, we did not identify the nuclear GLI-1 and Shh ligand in MF1, but Ihh expression was detected using immunocytochemistry (Figure 1C).

To evaluate the influence of IGF1R on the survival and proliferation of SCC-4 cells, we performed gene editing experiments with CRISPR/Cas9 to block IGF1R expression. After transfection, positive clones were selected by puromycin resistance, followed by subsequent evaluation of IGF1R expression by flow cytometry in the bulk population. In contrast to control cells exhibiting high IGF1R expression, gene-edited cells consisted of three populations: high IGF1R expression, knockdown and knockout (Figure S2). Cell sorting was conducted to enrich the population with low IGF1R expression, among which knockout cells originally represented 5.33% of total cells. Following sorting, this population represented 26.9% of total cells in culture. One week after sorting, the cells were again evaluated by flow cytometry; however, the IGF1R-negative population was no longer detected, suggesting that IGF1R is essential for SCC-4 survival (Figure S2). 

In order to evaluate whether stromal cells exert tumor-promoting activity through IGF-1, MF1 were genetically modified by a lentiviral system for IGF-1 overexpression, followed by characterization using RT-qPCR (Figure S3). 

**Figure 2:**
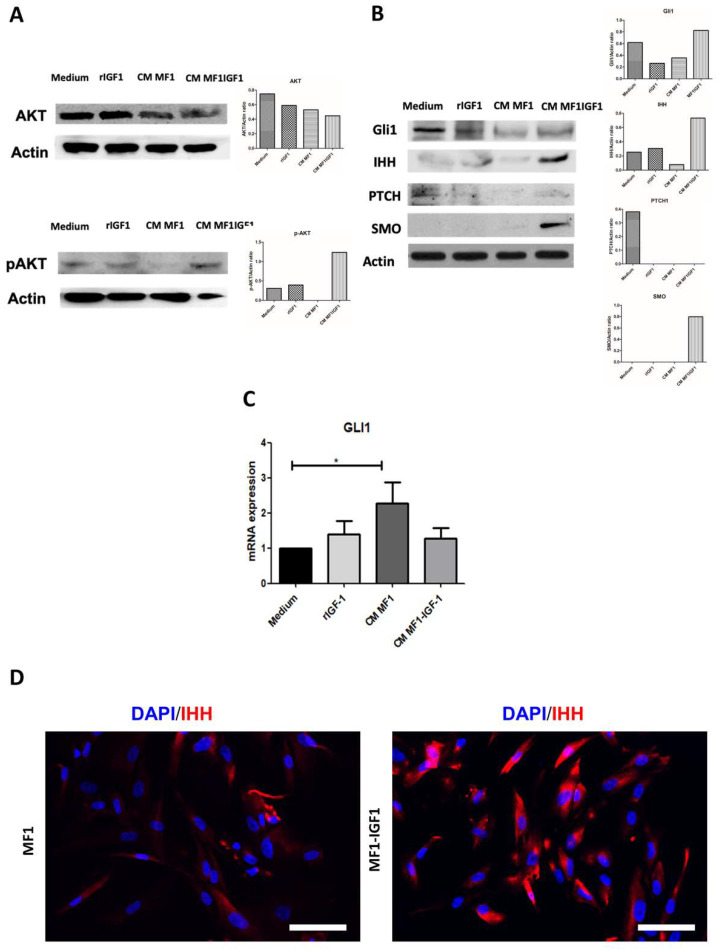
Regulation of AKT and HH pathways in SCC-4 by stimulation with IGF-1 or fibroblast-conditioned media. SCC-4 cells were stimulated for 24 h with 10% FBS, 100 ng/mL rIGF-1 or 50% conditioned medium from control fibroblasts (MF1) or cells overexpressing IGF-1 (MF1-IGF-1). Western blot analysis was performed to detect (**A**) total AKT and phospho-Akt (S473), with band intensity measured and plotted using ImageJ; (**B**) GLI1, IHH, PTCH1 and SMO band intensities were measured and plotted using ImageJ; (**C**) GLI1 mRNA expression was evaluated by RT-qPCR 6 h after stimulating cells with rIGF-1 or conditioned media. Data are presented as means ± SDs, bars represent comparisons between respective groups and (*) denotes statistical significance after applying the one-way ANOVA and Dunnett’s post-test, *p* < 0.05. CM: conditioned medium. (**D**) IHH immunostaining in MF1 and MF1-IGF-1 fibroblasts. The presence of the IHH ligand is shown in red, while nuclei were stained with DAPI (blue). Bars = 50 μm.

Stimulation of SCC-4 cells with either rIGF-1 or MF1-IGF-1 conditioned medium resulted in an increase in Akt phosphorylation, indicating similar activation of the PI3K pathway, which was not observed upon stimulation with MF1-conditioned medium (Figure 2A). The same samples were assessed for expression of HH pathway molecules by Western blot, which showed GLI1 expression in all conditions, and increased expression of IHH and SMO in SCC-4 cells stimulated with MF1-IGF-1 and SMO (Figure 2B). In contrast, qPCR demonstrated that GLI1 was not induced by MF1-IGF-1 nor rIGF-1 stimulation, but only by MF1-conditioned medium (Figure 2C). The presence of IHH was observed in both fibroblasts (MF1 and MF1-IGF-1), as shown in Figure 2D.

**Figure 3:**
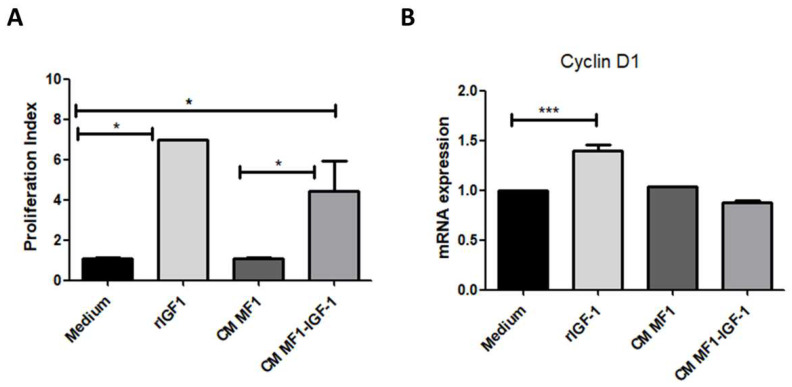
Cell proliferation analysis after stimulation of SCC-4 cells with IGF-1 or fibroblast-conditioned media. Control medium, rIGF-1, CM MF1 or CM MF1-IGF1 was used to stimulate SCC-4 cells. (**A**) After 72 h, proliferation analysis was performed by flow cytometry (CFSE assay). Analysis performed in quintuplicate, data presented as means ± SDs, bars represent comparisons between respective groups and (*) denotes statistical significance after applying the Mann–Whitney test < 0.05. (**B**) Cyclin D1 mRNA levels were assessed by RT-qPCR 6 h after incubation with stimuli. Results shown are representatives of three experiments each, data presented as means ± SDs, bars represent comparisons between respective groups, (*) denotes statistical significance after applying the one-way ANOVA and Dunnett’s post-test, *p* < 0.05 and (***) denotes statistical significance after applying the one-way ANOVA and Dunnett’s post-test, *p* < 0.01. CM: conditioned medium.

To assess the roles of MF1-conditioned media and IGF-1 on cell proliferation, SCC-4 cells were labeled with CFSE and incubated with rIGF-1 or conditioned media. We observed increased SCC-4 proliferation in the presence of IGF-1, both with the recombinant IGF-1 protein and with MF1-IGF-1 conditioned medium (Figure 3A). In addition, rIGF-1 also increased cyclin D1 mRNA expression (Figure 3B). 

**Figure 4:**
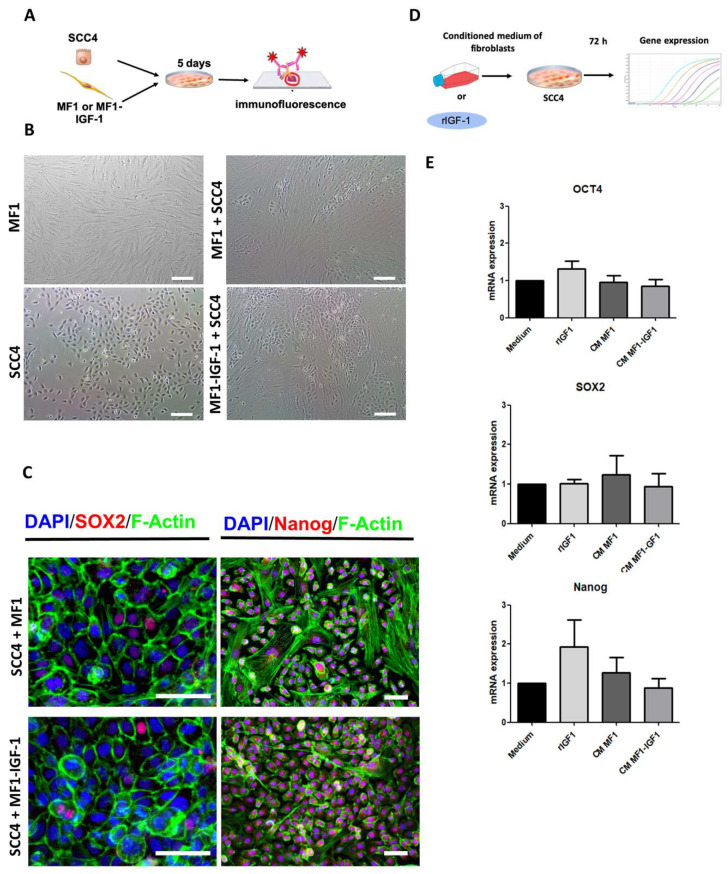
Evaluation of the expression of pluripotency markers in SCC-4. (**A**) Schematic design of the co-culture experiments. (**B**) Representative phase-contrast images of SCC-4/fibroblast co-culture. Bars = 100 μm. (**C**) Confocal microscopy images of cells stained for actin-F (green), pluripotency markers Sox-2 or Nanog (red) and nuclei stained with DAPI (blue). Differential cytoplasmic actin content and morphology allowed for the identification of fibroblasts as large cells with high expression of green fluorescence. Bars = 50 μm. (**D**) Schematic design of experiments evaluating pluripotency gene expression (OCT4, SOX2 and Nanog) by RT-qPCR following SCC-4 cell stimulation with control medium, rIGF-1, MF1-CM or MF1-IGF-1-CM for 72h. (**E**) Graphs detail the expression of each gene under different conditions. CM: conditioned medium.

To understand the roles of cell contact in the expression of pluripotency markers and GLI1 activation, SCC-4 was co-cultured with either MF1 or MF1-IGF-1. We observed that co-culture induced the organization of SCC-4 in islands surrounded by fibroblasts, resembling tumor organization (Figure 4B). The cells were then evaluated for GLI1 expression, along with pluripotency markers associated with populations of cancer stem cells. Regardless of culture conditions, pluripotency factors, such as SOX2, Nanog (Figure 4C) and GLI1 (Figure S4), were detected by immunofluorescence analysis.

SOX2, OCT4 and Nanog pluripotency genes were evaluated by RT-qPCR following tumor cell stimulation with rIGF-1 or conditioned media, with similar levels of transcripts found under all conditions and stimuli (Figure 4E).

**Figure 5:**
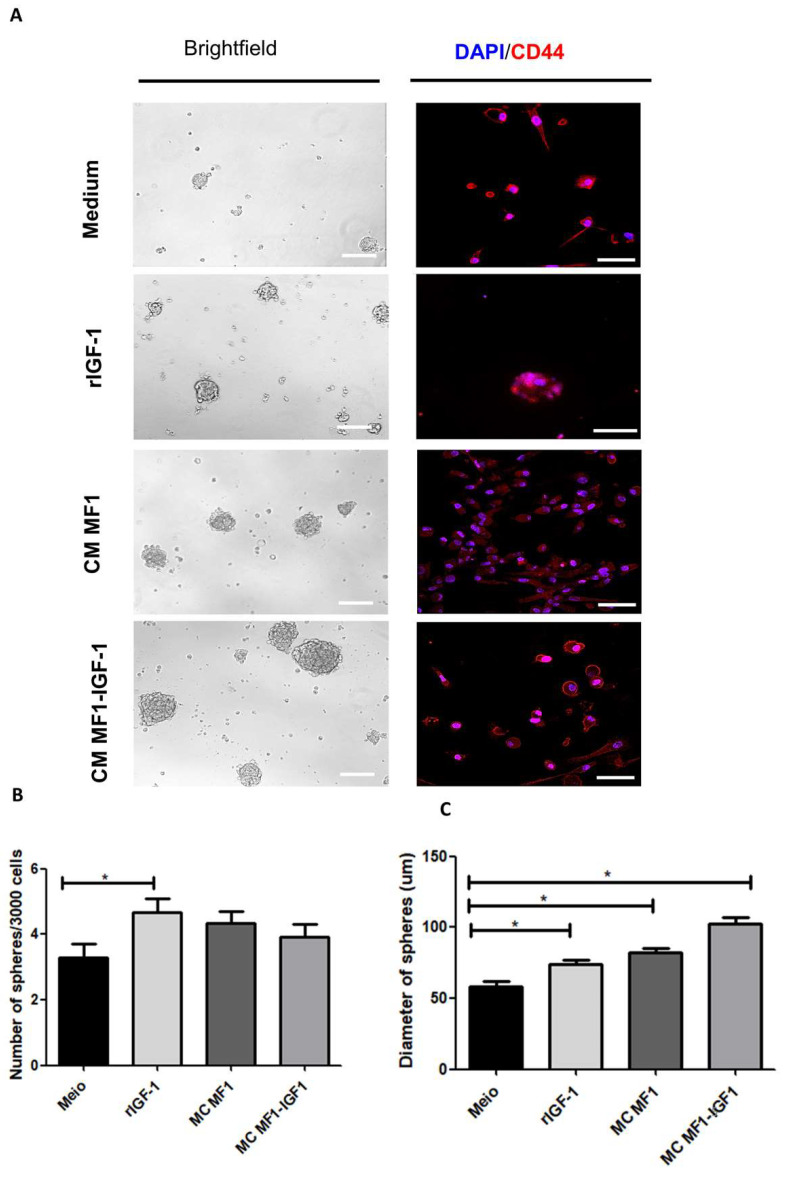
Tumorsphere formation assay. (**A**) Phase contrast images (left) showing morphologies of tumorspheres formed after 8 days under stimulation with rIGF-1 or conditioned media. Spheres were dissociated and immunostaining was performed (right) for detection of cancer stem cell marker CD44 (red) and DAPI for nuclei staining (blue). Bars: 200 μm (phase contrast images; left) and 50 μm (fluorescence images; right). (**B**,**C**) Quantification of the number (**B**) and diameter (**C**) of tumorspheres formed under different conditions. Data are presented as means ± SDs, bars represent comparisons between respective groups and (*) denotes statistical significance after applying the one-way ANOVA and Dunnett’s post-test, *p* < 0.05. CM: Conditioned medium.

The population of cancer stem cells was estimated by performing a tumorsphere-formation assay under different conditions (tumorsphere formation medium, rIGF-1 or fibroblast-conditioned medium). Subsequently, the number and diameter of the spheres formed under the different conditions were evaluated. A statistically significant increase in the number of spheres was found in SCC-4 stimulated with rIGF-1 compared to the sphere-formation medium (Figure 5B). Increased sphere size was observed in cells treated with rIGF-1 or conditioned media MF1-IGF-1, compared to sphere-formation medium (Figure 5C). Cancer stem cell marker CD44 was detected by immunofluorescence analysis of the cells derived from tumorspheres in all experimental conditions (Figure 5A). 

**Figure 6:**
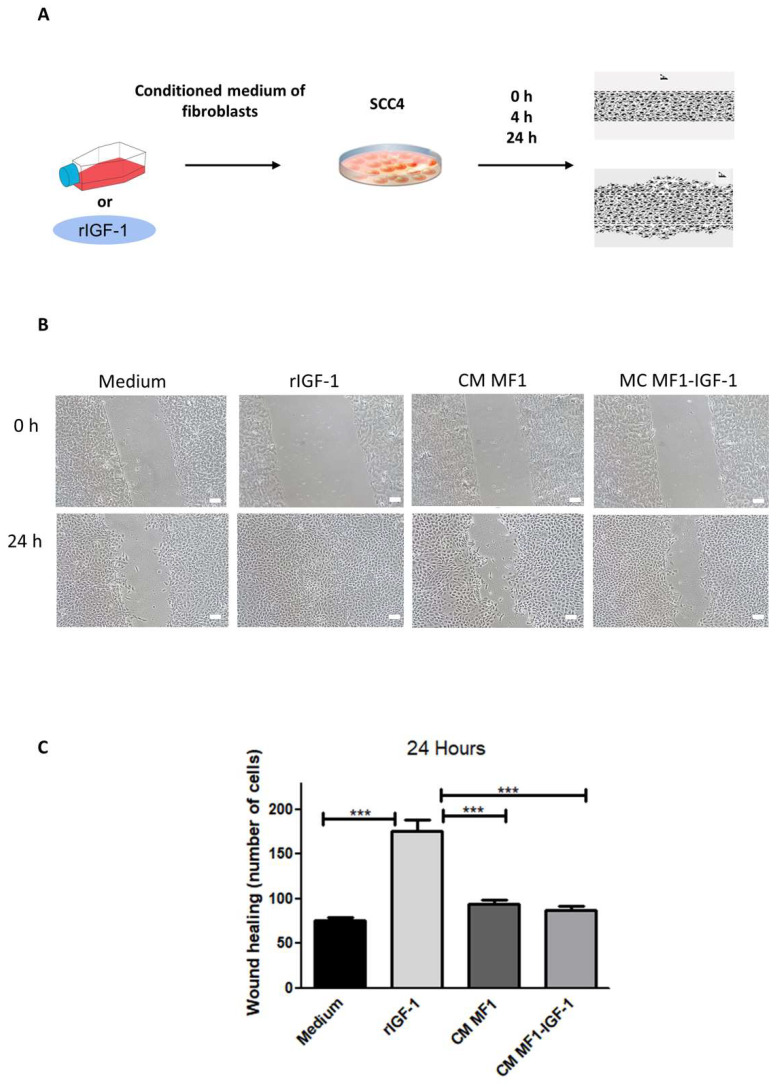
Evaluation of cell migration. (**A**) Experimental design of the scratch assay. (**B**) Representative images of cell migration at 0 and 24 h under different conditions. Bars: 50 μm. (**C**) Quantification of migrating cells. Data are presented as means ± SDs, bars represent comparisons between respective groups and (***) denotes statistical significance after applying the one-way ANOVA and Dunnett’s post-test, *p* < 0.01. CM: conditioned medium.

Cell migration was evaluated after incubation with the different stimuli (medium, rIGF-1 or fibroblast-conditioned medium) for 24 h. The assay demonstrated that only rIGF-1 was able to increase cell migration (Figure 6C). 

**Figure 7:**
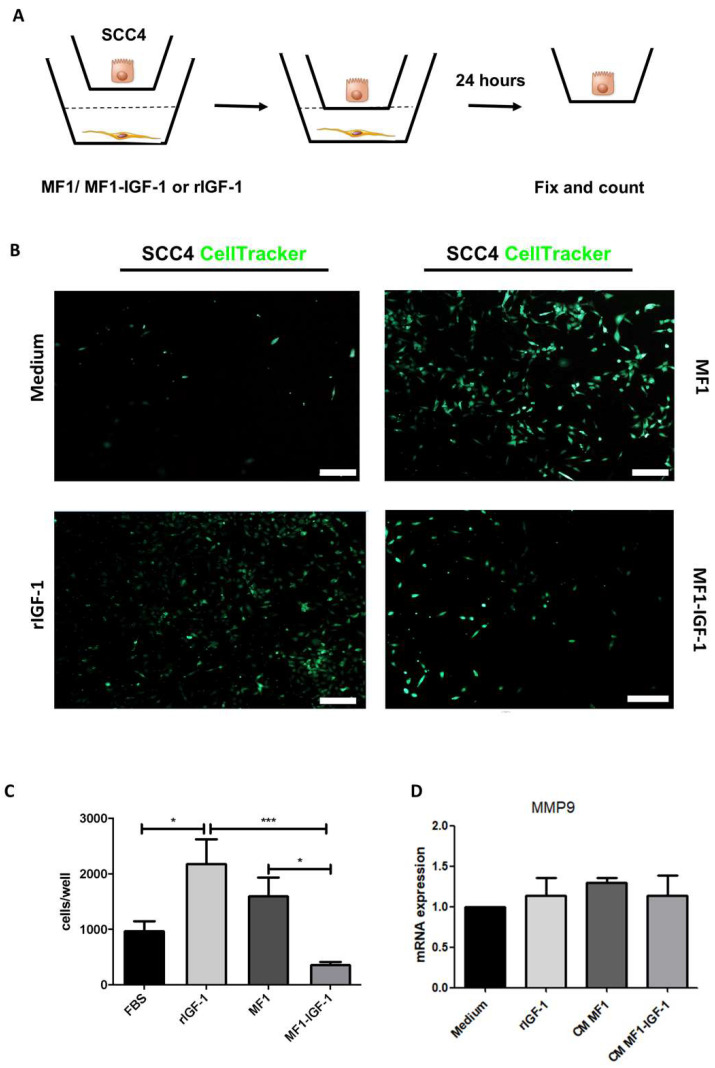
SCC-4 tumor cell invasion assay. (**A**) The invasiveness of SCC-4 cells was investigated by the Transwell assay, as represented by the schematic figure detailing experimental methodology. (**B**) CellTracker Green-stained SCC-4 cells are seen in the lower chamber at 24 h of incubation. Bars: 100 μm. (**C**) Quantification of cells invading the matrix. Results from seven replicas, data presented as means ± SDs, bars represent comparisons between respective groups, (*) denotes statistical significance after applying the one-way ANOVA and Dunnett’s post-test, *p* < 0.05 and (***) denotes statistical significance after applying the one-way ANOVA and Dunnett’s post-test, *p* < 0.01. (**D**) Quantification of matrix metalloproteinase 9 (MMP9) mRNA by RT-qPCR in SCC-4, 6 h after stimulation with rIGF-1, MF1-CM or MF1-IGF-1-CM. CM: conditioned medium.

To evaluate invasion, a transwell assay was performed by plating CellTracker-labeled SCC-4 in a geltrex-coated surface in the upper chamber and plating either MF1 or MF1-IGF-1 fibroblasts in the lower chamber. The effects of serum-free medium or medium supplemented with rIGF-1 in the lower chamber were also evaluated. We observed increased SCC-4 cells in the lower chamber in rIGF-1 and MF1-stimulated conditions (Figure 7C). The observed effects were not associated with any change in MMP9 mRNA expression, as demonstrated by RT-qPCR analysis (Figure 7D).

**Figure 8:**
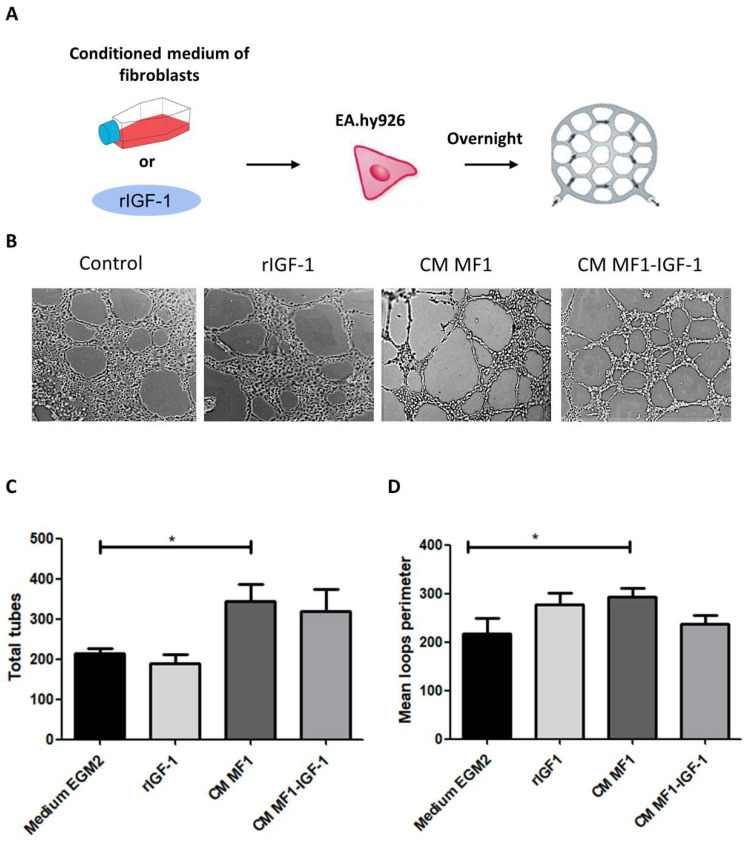
Endothelial tube formation assay. (**A**) Endothelial cells of the EA.hy927 strain were used in the angiogenesis assay, seeded under an artificial matrix (Geltrex) and incubated overnight with different stimuli (medium, rIGF-1, CM MF1 or CM MF1-IGF-1) as simplified in the scheme. (**B**) Representative images of capillary-like structures formed under each condition. Measurements of the total vessels, Magnification: 400×. (**C**) and loop perimeter (**D**) formed under different conditions. Result of six replicates, data presented as means ± SDs, bars represent comparisons between respective groups and (*) denotes statistical significance after applying the one-way ANOVA and Dunnett’s post-test, *p* < 0.05. CM: conditioned medium.

Finally, we evaluated the potential effects of rIGF-1 and conditioned media in the promotion of angiogenesis by performing a tube formation assay using immortalized endothelial cells EA.hy-926. Conditioned media from MF1 were associated with a larger number and perimeter of capillary-like structures (Figure 8C,D).

## 3. Discussion

The present study investigated the effects of IGF-1 on AKT and HH signaling pathways in OSCC, and its influence on pro-tumoral processes [18,19,20,21,22,23]. We observed that (i) IGF-1 exerts mitogenic actions, as evidenced by the increased proliferation index and transcriptional activity seen in the CCND1 gene; (ii) IGF-1 stimulates numbers and size of tumorsphere formation; (iii) rIGF-1 increases cell migration and invasion; (iv) angiogenesis was stimulated by MF1-conditioned medium; (v) rIGF-1 and conditioned media from both fibroblasts MF1 and MF1-IGF-1 led to AKT phosphorylation and GLI1 protein expression, yet only MF1-IGF-1-conditioned medium induced SMO accumulation; (vi) IHH protein expression was most evident in SCC-4 after stimulation with MF1-IGF-1-conditioned medium; (vii) only MF1-conditioned medium increased levels of GLI1 mRNA.

Proliferation studies showed that both rIGF-1 and MF1-IGF-1 conditioned medium promoted SCC-4 proliferation. In addition, only stimulation with rIGF-1 increased transcriptional levels of cyclin D1. It is possible, therefore, that increased proliferation may be mediated by different transcription factors and signaling pathways in cells stimulated with MF1-IGF-1, or even through differences in the kinetics of activation, which were not assessed herein. The more pronounced effect of rIGF-1 compared to IGF-1 secreted by MF1-IGF-1 can probably be explained by cytokine levels, and bioavailability. Secreted IGF-1 can complex with other proteins present in the conditioned medium, such as IGFBPs. A preliminary analysis of the conditioned media composition of MF1-IGF1 indicated upregulation of IGFBP7 (Figure S5), a protein that binds and reduces IGF-1 bioavailability [24,25,26,27,28,29]. 

IGF-1 plays critical roles in the control of apoptosis [30]. Osteosarcoma cells modified by IGF1R knock-in expressed increased Bcl-2, reduced Bax and had less cleavage of caspases 3 and 9 [31]. IGF1R expression has been related to both the intrinsic mitochondrial pathway, as observed in colon cancer cells [6], and the TNF-TRAIL pathway in melanoma cells [31]. In the current study, we silenced IGF1R using CRISPR/Cas9 in SCC-4. However, we were unable to maintain IGF1R knockout cells in culture, as was previously reported [32]. Even cells with mutations in the IGF1R sequence still maintained some level of receptor expression. These observations reinforce the critical role of IGF1R in cell survival.

Fibroblasts, the most abundant cell type found in tumor stroma, secrete several growth factors, including IGF-1, and cytokines, chemokines and metalloproteinases [33,34]. CAFs participate in angiogenesis, migration, invasion, proliferation and the regulation of signaling pathways, which contributes to tumor severity. In OSCC samples, the presence of CAFs is related to a worse prognosis [35,36]. Initially, we investigated whether fibroblast-secreted IGF-1 provokes non-canonical hedgehog (HH) pathway activation. Interest in this signaling pathway is justified by previous studies from our group that reinforce the importance of HH activity in OSCC tumorigenesis [37,38]. In addition, our recent results have shown that some OSCC cell lines are unresponsive to the ligands that perform canonical HH activation (SHH, for example), suggesting the involvement of other signaling pathways in the activation of GLI1 transcripts. Bilateral communication between IGF-1 and GLI1 has been observed in some tumors; i.e., IGF-1 may increase GLI1 activity, while HH targets may in turn increase IGF-1 expression [39,40,41,42]. However, in the context of OSCC, these types of relationships have not yet been fully elucidated.

The present work evaluated GLI1 in SCC-4 by qPCR, immunofluorescence and Western blot. Only stimulation with MF1-conditioned medium was found to increase GLI1 mRNA levels in SCC-4. On the other hand, GLI1 fluorescence intensity remained unaltered when co-culturing MF1 and SCC-4 cells. Importantly, this effect was confirmed by neither Western blot nor immunofluorescence. The expression of other HH pathway molecules was also evaluated by Western blot (PTCH1, SMO and IHH) in these same samples, and only MF1-IGF-1 fibroblast-conditioned medium led to SMO expression. In addition, this stimulus was also found to be the most potent with regard to IHH expression. Accordingly, we speculate that different stimuli influence GLI1 expression through distinct signaling axes, which occur independently of SMO. 

Given that IGF-1 can activate GLI1 by PI3K-AKT [43], we proceeded with the evaluation of AKT activity in the same samples in which GLI1 expression was observed. We hypothesized that rIGF-1/IGF1R interaction transmits intracytoplasmic signals that result in AKT phosphorylation, which in turn activate GLI1 transcription [43]. The presence of IHH expression in both MF1 and MF1-IGF-1 could likely be a component of fibroblast-conditioned medium. Accordingly, MF1-secreted IHH may bind to the PTCH1 receptor and activate GLI1 independent of SMO. It follows that GLI1 transcriptional activity can promote AKT phosphorylation, which in turn contributes to higher GLI1 expression [44]. MF1-IGF-1 secreted IHH couples to the PTCH1 receptor, which transmits signaling to SMO, and in turn activates intracellular cascades, resulting in further GLI1 expression. At the same time, IGF-1 (also fibroblast-secreted) activates GLI1 via the IGF1R/AKT/GLI1 intracytoplasmic pathway. In this context, in addition to the observed GLI1 expression, transcriptional activity of target genes in the HH pathway, such as IHH, may be explained by the greater stability of the transcription factor GLI1 under the condition of mutual activation of the AKT and HH pathways [45,46].

The establishment and maintenance of cell stemness are important for maintaining tumor growth rates, which are mediated by increased proliferation and self-renewal, and the inhibition of apoptosis. These characteristics of cancer stem cells result in resistance to therapy and tumorigenicity in in vivo models [47]. Different cell markers have been proposed for the definition of cancer stem cells in OSCC, including CD44, CD133, ALDH, cMET and GRP78 [48]. Moreover, cancer stem cells are able to form spheres in vitro [49,50]. The tumorsphere formation assay is used to determine the self-renewal capacity of tumor cells and is also currently employed to screen new anticancer drugs [51,52]. In the present study, we performed an SCC-4 tumorspheres assay, maintained against different stimuli for eight days in non-adherent plates. We sequentially immunostained with CD44 [48] which was positive for the spheres formed under all cultured conditions. However, more spheres formed in the presence of rIGF-1, with the largest diameters found in spheres stimulated with MF1-IGF-1-conditioned medium. On the other hand, under 2D cell growth conditions, no change was observed in mRNA levels of OCT4, SOX2 and Nanog, nor in the immunostaining of SOX2 and Nanog in SCC-4 co-cultured with fibroblasts. These data reinforce the relevance of the sphere formation assay for the study of tumor stem cells, and the importance of IGF-1 as a stimulus for tumor stem cell maintenance [53].

In addition to tumor stem cells, some authors believe that IGF-1 is also an important stimulus for angiogenesis [54]. However, in our study, no significant influence of IGF-1 on vessel formation was observed. The largest number and vessel perimeters were found in the stimulation condition with wild fibroblasts conditioned medium. This observation, on the other hand, demonstrates the participation of fibroblasts’ secretome in the tumor microenvironment in pro-tumorigenic events, such as angiogenesis [55]. 

Although IGF-1 did not have a significant effect on angiogenesis in relation to migration and invasion, rIGF-1 was identified as the most important stimulus. Our observations corroborate previous studies showing the participation of IGF-1 in tumor cell migration in multiple models of myeloma [56], hepatocellular carcinoma [57] and melanoma [58]. The possible mechanism underlying IGF-1-induced SCC-4 migration may be via the IGF1R/AKT axis or by IGF-1 itself [59].

Invasion can also be triggered by activation of the IGF1R/AKT axis, as shown in pancreatic tumor cells [60]. rIGF-1 activated AKT in our study, as it was the stimulus that induced the greatest invasion of SCC-4 cells in the artificial matrix (Geltrex). The greater degradation of extracellular matrix by AKT-activated cells may be justified by the activation of matrix metalloproteinases [61]. However, MMP9 expression was demonstrated to not be induced after 6h of stimulation with rIGF-1. It is possible that the time in which MMP9 transcriptional activity was evaluated was not compatible with the time elapsed in the process of cell invasion by the transwell assay (24 h). 

In summary, this study describes the action of IGF-1 on tumor proliferation, migration, invasion and the maintenance of stemness in SCC-4, which shows high IGF-1 receptor expression but does not synthesize this growth factor. Additionally, the importance of conditioned fibroblast media for angiogenesis was observed. Both fibroblast-conditioned-medium and rIGF-1 controlled the activation of HH and AKT pathways, which opens up prospects for investigating the simultaneous blockading of both pathways as a therapeutic target in OSCC [62].

## 4. Materials and Methods 

### 4.1. Cell Culture and Characterization 

Human dermal fibroblasts (MF1) were previously obtained from a skin biopsy of a healthy male donor [63]. OSCC cell line SCC-4 (ATCC^®®^ CRL1624™, Manassas, VA, USA) was used in all experiments after a previous screening to confirm the presence of hedgehog pathway components (Shh, PTCH1, SMO and GLI1). In addition Brady et al., (2007) [64] evaluated the expression of IGF1R and IGF-1 in different tumor cell lines (SCC-4, SCC-25, FaDu and TR146) and these authors found a high expression of IGF-1 receptor, but not the IGF-1 ligand, in the SCC-4 cell line. We confirmed these results through flow cytometry and gene expression (Figure 1A,B). Thus, by performing the experiments with SCC-4 cells, we eliminated the autocrine stimulation bias that could be caused by IGF-1 secreted by the tumor cell.

MF1 and SCC-4 were cultured in DMEM supplemented with 10% FBS and 1% penicillin/streptomycin (all from Thermo Fisher Scientific, Waltham, MA, USA), at 37 °C in a 5% CO_2_ humidified atmosphere. The cells were assessed for IGF-1 production and IGF1R expression by RT-qPCR (see PCR section) and flow cytometry analysis, respectively. 

For flow cytometry analysis, the cells were trypsinized, counted and labeled according to the manufacturer’s recommendation with anti-IGF1R antibody (CD221-APC #17-8849-42, Thermo Fisher Scientific, Waltham, MA, USA). Unstained cells were used as controls. At least 10,000 events were acquired in a flow cytometer (BD Fortessa, West Lafayette, IL, USA).

### 4.2. Genetic Modification

In order to induce IGF-1 overexpression, MF1 were transduced with lentiviral vectors, as previously described [65]. Briefly, 2 × 10^5^ MF1 cells at passage 9 were incubated for 24 h with 6 µg/mL polybrene (Sigma Aldrich, St. Louis, MO, USA) and 2nd generation lentiviruses containing the human IGF-1 sequence (MOI 10). After 24 h, medium was changed and stable clones were selected by the addition of puromycin (2 μg/mL; Sigma Aldrich, St. Louis, MO, USA) to the culture medium. As a transduction control, we utilized pEGIP (Addgene #26777, Watertown, MA, USA), which encodes green fluorescent protein (GFP) and puromycin resistance genes. Mock-transduced MF1 cells were used as negative controls during puromycin selection.

IGF1R gene silencing was performed in SCC-4 cells by CRISPR/Cas9 gene-editing utilizing a lentiviral system with a previously validated sgRNA sequence [66] (Addgene #76689, Watertown, MA, USA). Lentiviral particles were prepared by transfection of HEK293FT cells with the sgRNA vector, psPAX2 (Addgene #12260, Watertown, MA, USA) and pMD2.G (Addgene #12259, Watertown, MA, USA) in the ratio 3:2:1, as previously described [21]. Subsequently, 2 × 105 cells were transduced with Cas9 expression vector, as described above, and after 48 h, transduced cells were selected by incubation with 10 μg/mL blasticidin (Thermo Fisher Scientific, Waltham, MA, USA). After expanding the blasticidin-resistant population, another round of lentiviral transduction was performed with lentivirus coding for the sgRNA targeting IGF1R and subsequent selection with 2 μg/mL puromycin (Sigma Aldrich, St. Louis, MO, USA). SCC-4 cells were then assessed for IGF1R expression by flow cytometry.

### 4.3. MF1 Conditioned Media

To obtain conditioned media from MF1 and fibroblasts overexpressing IGF-1 (MF1-IGF1), cells were cultured in growth medium until 80% confluency was achieved. Then, the complete medium was removed, the wells were washed with PBS, and DMEM serum-free was added to the flask. MF1 and MF1-IGF1 were maintained in starvation for 72 h when the conditioned media was harvested and centrifuged at 3500× *g* for 10 min for removal of cell debris. The supernatant was then collected, aliquoted and stored at minus 80 °C until use.

### 4.4. Gene Expression Analysis by RT-qPCR

For RT-qPCR reactions, the cells were detached using Trypsin (Thermo Fisher Scientific, Waltham, MA, USA) and RNA was extracted with Rneasy Plus Mini Kit (QIAGEN, Frederick, MD, USA), following the manufacturer’s instructions. Reverse transcription reactions to obtain cDNA were performed from 2.5 μg/mL of total RNA using Superscript VILO™ (Invitrogen Corporation, Carlsbad, CA, USA) according to the manufacturer’s protocol. The reactions were incubated at 25 °C for 10 min, 37 °C for 120 min and 85 °C for 5 s. The following genes (TaqMan™ Gene Expression Assays, Applied Biosystems™, Foster City, CA, USA)were evaluated: GLI1 (Hs01110766_m1), POU5F1 (Hs00999632_G1), SOX2 (Hs0060273000_S1), NANOG (#Hs02387400_G1), CCND1 (#Hs00765553_m1), MMP9 (#Hs00234579_m1) and IGF-1 (#Hs01547656_m1). After testing with an endogenous gene panel, B2M (#Hs99999907_m1) was chosen as the normalizer.

All reactions were conducted in the ABI ViiA7 (Applied Biosystems™, Foster City, CA, USA) apparatus using 96-well Fast plates with a total volume of 20 μL. Each well consisted of 2.5 ng/μL sample cDNA (4 μL), 1 μL Assay (Applied Biosystems, Foster City, CA, USA), 10 μL FAST Taqman PCR Gene Expression Master Mix Kit (Applied Biosystems, Foster City, CA, USA) and 5 μL water RNAse free. The amplification program consisted of an initial cycle of 50 °C for 2 min and 95 °C for 10 min, followed by 40 cycles of 95 °C for 15 s and 60 °C for 1 min.

After amplification runs, quantification cycle (Cq) values were provided by the Viia7™ System SDS Operational Program (Applied Biosystems, Foster City, CA, USA). The Cq value of each sample was normalized using the geometric mean Cq value of the reference gene, corresponding to ∆CT. Then, the ∆∆CT was calculated, which refers to the Cq of the sample of interest, subtracted from the ∆CT of the unstimulated cell (cell in medium). Finally, the value of 2-∆∆Ct was considered.

### 4.5. Immunostaining 

Immunocytochemistry of MF1 fibroblasts was performed for the identification of HH pathway molecules (SHH, IHH, PTCH1 and GLI1). Following incubation with primary antibodies, HRP Link and HRP Enzyme reagents (Advance™, Dako Corporation, Carpinteria, CA, USA) were applied to the coverslips, 20 min each, followed by incubation with 3,3-diaminobenzidine for 5 min (Dako, Carpinteria, CA, USA). Immunofluorescence of MF1 and MF1-IGF1 cultured in the presence of DMEM supplemented with 10% FBS was performed to evaluate the expression of IHH in both strains. 

For immunofluorescence, the wells were washed with PBS, followed by fixation with 4% paraformaldehyde for 20 min and the permeabilization was performed by incubation with Triton-X100 (Sigma-Aldrich, St. Louis, MO, USA). After washing, a protein block was performed, followed by overnight incubation with primary antibodies at 4 °C. The following primary antibodies were used: GLI1 (1:500; Novus #NB800, Centennial, CO, USA), PTCH1 (1:500; Novus #NB200-118 Centennial, CO, USA), SHH (1:500; Novus #NBP2-22126, Centennial, CO, USA), IHH (1:500; Abcam #EP1192, Branford, CT, USA), pan-cytokeratin (1:500; MyBiosource IML-91, San Diego, CA, USA), Nanog (1:200; Santa Cruz SC1732, Dallas, TX, USA) and SOX2 (1:200; Millipore AB5731, Darmstadt, Alemanha). On the following day, incubation with the secondary antibodies donkey anti-mouse IgG Alexa Fluor 647 1:1000 (Thermo Fisher A-32787, Waltham, MA, USA), rabbit anti-goat IgG Alexa Fluor 568 1:1000 (Thermo Fisher A-11079, Waltham, MA, USA) and goat anti-rabbit IgG Alexa Fluor 488 1:600 (Thermo Fisher A-11008, Waltham, MA, USA), was performed for 1 h at RT. Nuclei were counterstained with DAPI (Vector Labs, Burlingame, CA, USA). For the validation of the tumorspheres formation assay, spheres and SCC-4 cells in 2D culture conditions were labeled with an anti-CD44 Alexa Fluor 647 (1:100 BD Biosciences 550989, West Lafayette, USA). At the end of immunostaining, the nuclei were stained with DAPI (Vector Labs, Burlingame, CA, USA). Images were acquired with confocal A1+ microscopy (Nikon, Tokyo, Japan) or with the Operetta High Content Imaging System (Perkin Elmer, Waltham, MA, USA).

### 4.6. Cell Proliferation Assay 

Cell proliferation was assessed by flow cytometry using SCC-4 labeled with Carboxyfluorescein succinimidyl ester (CFSE) (Cell Tracker Green, Thermo Scientific, Waltham, MA, USA) following the manufacturer’s recommendations. Labeled cells (5 × 105) were incubated in the presence of rIGF-1 (100 ng/mL, Thermo Scientific, Waltham, MA, USA) or 50% conditioned medium (MF1 or MF1-IGF-1) and 50% DMEM (Thermo Fisher Scientific, Waltham, MA, USA) supplemented with 10% FBS for 72 h at 37 °C and 5% CO_2_. After the incubation period, the cells were trypsinized and centrifuged. The pellet was resuspended in PBS and 1% formaldehyde and analyzed by flow cytometry acquisition (BD Fortessa, West Lafayette, IL, USA); 10,000 events were acquired, followed by data analysis using Flowjo version 7.6 software (BD, West Lafayette, IL, USA). For data analysis, the proliferation index was used. 

### 4.7. Scratch Assay

SCC-4 cells were plated in 12-well plates (2.5 × 104 cells/well) and were maintained in DMEM+ 10% FBS and 1% Penicillin/Streptomycin until a 100% confluent monolayer was obtained. Then, the cells were kept in starvation for 24 h. In order to inhibit cell proliferation, mitomycin C (12 μg/mL Sigma Aldrich, St. Louis, MO, USA) was added for 2 h, wells were washed with saline and serum-free DMEM medium (Thermo Fisher Scientific, Waltham, MA, USA) containing the stimuli (100 ng/mL of rIGF-1, 50% MF1 or MF1-IGF1 conditioned medium or culture medium) was added to the wells. In the center of each well, a scratch was made with a 200 μL tip. The wells were photographed at 0, 4 and 24 h. Ten images from each well were taken at each timepoint for quantification of migrating cells NIS-elements (Nikon, Tokyo, Japan) software. 

### 4.8. Invasion Assay

The invasiveness of SCC-4 cells after incubation with different stimuli was evaluated by a transwell invasion assay. SCC-4 cells were stained with Cell Tracker Green (Thermo Fisher Scientific, Waltham, MA, USA), and 4 × 104 cells/mL were plated in DMEM ( Thermo Fisher Scientific, Waltham, MA, USA) serum-free in the upper chamber, on a membrane coated with geltrex (Thermo Fisher Scientific, Waltham, MA, USA). In the lower chamber, MF1 or MF1-IGF-1 fibroblasts were plated (4 × 104 cells/well). The effect of serum-free DMEM supplemented with 100 ng/mL with rIGF-1 was also evaluated. After incubation for 24 h at 37 °C, the insert was removed, the membrane was fixed with 4% formaldehyde and the nuclei were stained with Hoechst 33342 (Thermo Fisher Scientific #1874027, Waltham, MA, USA). The number of cells that invaded the membrane was quantified from images obtained with the Operetta High Content Imaging System (Perkin Elmer, Waltham, MA, USA). 

### 4.9. Tube Formation Assay

In order to evaluate the potential of different stimuli (rIGF-1, MF1 or MF1-IGF-1 conditioned medium) to promote angiogenesis, endothelial cells of the EA.hy926 line (ATCC^®®^ CRL-2922) were used in a tube formation assay. Geltrex (Thermo Fisher Scientific, Waltham, MA, USA) was added to wells of a 96-well plate (40 µL/well) and allowed to polymerize by incubation at 37 °C for 30 min. EA.hy926 cells were then plated (4 × 104 cells/well), and maintained with the following media: (i) EGM2-MV medium (positive control); (ii) DMEM medium (2% SFB and 1% penicillin/streptomycin) containing 100 ng/mL rIGF-1; (iii) DMEM (2% SFB and 1% penicillin/streptomycin) with 50% MF1-CM; and (iv) DMEM (2% SFB and 1% penicillin/streptomycin) with 50% MF1-IGF-1-CM.

The cells were incubated overnight at 37 °C and 5% CO_2_. The images were then acquired with the Operetta High Content Imaging System (Perkin Elmer, Waltham, MA, USA). Thirty images were obtained from each well and used for automatic quantification with the Tube Formation ACAS Image Analysis (IBIDI, Gräfelfing, Germany) online tool. Tube characteristics, such as extension, number of branches, total communications and interlacing points were evaluated by the software. 

### 4.10. Tumorsphere Formation Assay

Tumorspheres were allowed to form by the plating of 3000 SCC-4 cells in ultra-low adhesion flat-bottom 96-well plates (Corning, Glendale, AZ, USA) for 8 days in tumorsphere medium (DMEM/F12 supplemented with 1 × B27, 20 ng/mL EGF, 10 μg/mL insulin and 1% penicillin/streptomycin). Addition of 100 ng/mL of rIGF-1 or 50% conditioned medium (MF1 or MF1-IGF-1) was evaluated and compared to control tumorsphere medium. The assay was performed in sexuplicate, and the growth of the tumorspheres was monitored under microscope visualization, images were acquired and quantification of the diameter and number of spheres was performed using the NIS-elements software (Nikon, Tokyo, Japan). 

### 4.11. Western Blot

The cell lysate was obtained with NE-PER™ Nuclear and Cytoplasmic Extraction Reagents (#78833, Thermo Fisher Scientific, Waltham, MA, USA), using the manufacturer’s protocol. The cytoplasmic proteins were fractionated on Bolt Bis-Tris Plus Gel polyacrylamide gel (Thermo Fisher Scientific, Waltham, MA, USA) and gel proteins were transferred to nitrocellulose membranes (BIO-RAD, Hercules, CA, USA) using the semi-dry method on a BIO-RAD trans-blot apparatus (BIO-RAD, Hercules, CA, USA) following the recommendations of the manufacturer. Following protein transfer, nonspecific binding sites of the membrane were blocked by incubation with 5% skim milk (*m/v*) in Tris-buffered saline (TBS, 137 mM NaCl, 24.8 mM Tris, pH 7.5) and 0.05% Tween 20 (*v/v*, TBS-T) for 1 h. At the end of the incubation period, the membranes were washed with TBS-T and the primary antibodies (1:500 anti-mouse-AKT R&D #281046, Minneapolis, MN, USA 1:250 anti-rabbit-pAKT R&D #AF887, Minneapolis, MN, USA 1:500 anti-rabbit-GLI1 Novus #NB800, Centennial, CO, USA 1:500 anti-rabbit-PTCH1 Novus #NB200-118, Centennial, CO, USA 1:500 anti-rabbit-IHH ABCAM #EP1192Y, Cambridge, MA, USA 1:200 anti-rabbit-SMO Santa Cruz #SC-13943 1:1000 anti-mouse-actin, Dallas, TX, USA) were added for 16 h. Later the membrane was washed with TBS-T3% skim milk and incubated 1 h with secondary antibodies (anti-mouse DAKO #HRP P0448, Santa Clara, CA, USA or anti-rabbit DAKO #HRP P0447, Santa Clara, Dallas, TX, USA). Then, the membrane was revealed by the Chemiluminescence Reagent kit (Abcam #ab79907, Cambridge, MA, USA).

### 4.12. Statistical Analysis

One-way ANOVA and Dunnett’s post-test were used to compare variances in independent groups A non-parametric Mann–Whitney test was used for proliferation assays. All statistical analyses were performed using GraphPad Prism version 4.01 (GraphPad Software). *p* < 0.05 (*) or *p* < 0.01 (***) were considered significant.

## Supplementary Materials

Supplementary materials can be found at https://www.mdpi.com/1422-0067/21/18/6487/s1.

## Abbreviations

OSCC	Oral squamous cell carcinoma
IGF1R	Insulin-like growth factor 1 receptor
PI3KCA	Phosphatidylinositol 3-kinase
AKT-PKB	Protein kinase B
RAS/RAF/MAPK	Mitogen-activated protein kinases pathway
TCGA	The Cancer Genome Atlas
PRKCI	Protein kinase C iota
GLI	Glioma-associated oncogene
IGF-1	Insulin-like growth factor 1
HH	Hedgehog
IL	Interleukin
CAF	Cancer-associated fibroblasts
TGF-β	Transforming growth factor beta
MMP	Matrix metallopeptidases
HGF	Hepatocyte growth factor
EGF	Epidermal growth factor
CTGF	Connective tissue growth factor
SMO	Smoothened
SHH	Sonic hedgehog
PTCH1	Patched 1
IHH	Indian hedgehog
IGFBP	Insulin-like growth factor-binding protein
MF1	Human dermal fibroblasts
MF1-IGF1	Human dermal fibroblasts-expressing IGF-1
TNF-TRAIL	Tumor necrosis factor-related apoptosis-inducing ligand
CRISPR/Cas9	clustered regularly interspaced short palindromic repeats/CRISPR associated protein 9
CD	Cluster differentiation
MOI	Multiplicity of infection
sgRNA	single guide RNA
DMEM	Dulbecco’s modified Eagle’s medium
CM	Conditioned medium

## References

[1] Sarradin V., Siegfried A., Uro-Coste E., Delord J.P. (2018). Classification de l’OMS 2017 des tumeurs de la tête et du cou: Principales nouveautés et mise à jour des méthodes diagnostiques. Bull. Cancer.

[2] Siegel R.L., Miller K.D., Jemal A. (2018). Cancer statistics, 2018. CA Cancer J. Clin..

[3] Foulstone E., Prince S., Zaccheo O., Burns J., Harper J., Jacobs C., Hassan A. (2005). Insulin-like growth factor ligands, receptors, and binding proteins in cancer. J. Pathol..

[4] Laviola L., Natalicchio A., Giorgino F. (2007). The IGF-I Signaling Pathway. Curr. Pharm. Des..

[5] Rabiee A., Krüger M., Ardenkjær-Larsen J., Kahn C.R., Emanuelli B. (2018). Distinct signalling properties of insulin receptor substrate (IRS)-1 and IRS-2 in mediating insulin/IGF-1 action. Cell. Signal..

[6] Wang Y., Jia L., Wang B., Diao S., Jia R., Shang J. (2019). MiR-495/IGF-1/AKT signaling as a novel axis is involved in the epithelial-mesenchymal transition of oral squamous cell carcinoma. J. Oral Maxillofac. Surg..

[7] Hao Y., Zhang C., Sun Y., Xu H. (2019). Licochalcone A inhibits cell proliferation, migration, and invasion through regulating the PI3K/AKT signaling pathway in oral squamous cell carcinoma. Onco Targets Ther..

[8] Li L., Li J.C., Yang H., Zhang X., Liu L.L., Li Y., Fu L. (2018). Expansion of cancer stem cell pool initiates lung cancer recurrence before angiogenesis. Proc. Natl. Acad. Sci. USA.

[9] Chen W.J., Ho C.C., Chang Y.L., Chen H.Y., Lin C.A., Ling T.Y., Pan S.H. (2014). Cancer-associated fibroblasts regulate the plasticity of lung cancer stemness via paracrine signalling. Nat. Commun..

[10] Cerami E., Gao J., Dogrusoz U., Gross B.E., Sumer S.O., Aksoy B.A., Schultz N. (2012). The cBio Cancer Genomics Portal: An Open Platform for Exploring Multidimensional Cancer Genomics Data: Figure 1. Cancer Discov..

[11] Gao J., Aksoy B.A., Dogrusoz U., Dresdner G., Gross B., Sumer S.O., Sun Y., Jacobsen A., Sinha R., Larsson E. (2013). Integrative Analysis of Complex Cancer Genomics and Clinical Profiles Using the cBioPortal. Sci. Signal..

[12] Owusu-Brackett N., Shariati M., Meric-Bernstam F. (2019). Predictive Biomarkers in Oncology.

[13] Lyra S.M.D.C., Moleri A.B., Dezonne R.S., Lourenço S.D.Q.C., Moura-Neto V., Pereira C. (2020). Evaluation of micrornas related to the sonic hedgehog pathway in oral cancer. Oral Surg. Oral Med. Oral Pathol. Oral Radiol..

[14] Takabatake K., Shimo T., Murakami J., Anqi C., Kawai H., Yoshida S., Nagatsuka H. (2019). The Role of Sonic Hedgehog Signaling in the Tumor Microenvironment of Oral Squamous Cell Carcinoma. Int. J. Mol. Sci..

[15] Kalluri R. (2016). The biology and function of fibroblasts in cancer. Nat. Rev. Cancer.

[16] De Palma M., Biziato D., Petrova T.V. (2017). Microenvironmental regulation of tumour angiogenesis. Nat. Rev. Cancer.

[17] Liao Z., Tan Z.W., Zhu P., Tan N.S. (2018). Cancer-associated fibroblasts in tumor microenvironment—Accomplices in tumor malignancy. Cell. Immunol..

[18] Javed S., Bhattacharyya S., Bagga R., Srinivasan R. (2020). Insulin growth factor-1 pathway in cervical carcinoma cancer stem cells. Mol. Cell. Biochem..

[19] Sun L., Yuan W., Wen G., Yu B., Xu F., Gan X., Tang J., Zeng Q., Zhu L., Zhang W. (2020). Parthenolide inhibits human lung cancer cell growth by modulating the IGF-1R/PI3K/Akt signaling pathway. Oncol. Rep..

[20] Peng Y., Li F., Zhang P., Wang X., Shen Y., Feng Y., Jia Y., Zhang R., Hu J., He A. (2020). IGF-1 promotes multiple myeloma progression through PI3K/Akt-mediated epithelial-mesenchymal transition. Life Sci..

[21] Wang L., Song Y., Wang H., Liu K., Shao Z., Shang Z. (2020). MiR-210-3p-EphrinA3-PI3K/AKT axis regulates the progression of oral cancer. J. Cell. Mol. Med..

[22] Chetty R. (2020). Gene of the month: GLI-1. J. Clin. Pathol..

[23] Tamayo-Orrego L., Gallo D., Racicot F., Bemmo A., Mohan S., Ho B., Salameh S., Hoang T., Jackson A.P., Charron F. (2020). Sonic hedgehog accelerates DNA replication to cause replication stress promoting cancer initiation in medulloblastoma. Nat. Cancer.

[24] Lim K.P., Cirillo N., Hassona Y., Wei W., Thurlow J.K., Cheong S.C., Prime S.S. (2011). Fibroblast gene expression profile reflects the stage of tumour progression in oral squamous cell carcinoma. J. Pathol..

[25] Oh Y., Nagalla S.R., Yamanaka Y., Kim H.S., Wilson E., Rosenfeld R.G. (1996). Synthesis and characterization of insulin-like growth factor-binding protein (IGFBP)-7 Recombinant human mac25 protein specifically binds IGF-I and-II. J. Biol. Chem..

[26] Annunziata M., Granata R., Ghigo E. (2011). The IGF system. Acta Diabetol..

[27] Evdokimova V., Tognon C.E., Benatar T., Yang W., Krutikov K., Pollak M., Sorensen P.H., Seth A. (2012). IGFBP7 binds to the IGF-1 receptor and blocks its activation by insulin-like growth factors. Sci. Signal..

[28] Wang Z., Wang Z., Liang Z., Liu J., Shi W., Bai P., Lin X., Magaye R., Zhao J. (2013). Expression and clinical significance of IGF-1, IGFBP-3, and IGFBP-7 in serum and lung cancer tissues from patients with non-small cell lung cancer. Onco Targets Ther..

[29] Bartram I., Erben U., Ortiz-Tanchez J., Blunert K., Schlee C., Neumann M., Heesch S., Baldus C.D. (2015). Inhibition of IGF1-R overcomes IGFBP7-induced chemotherapy resistance in T-ALL. BMC Cancer.

[30] Baserga R., Peruzzi F., Reiss K. (2003). The IGF-1 receptor in cancer biology. Int. J. Cancer.

[31] Xiao J., He C., Zhong Y., Liu L., Xiao Y., Liu W. (2019). miR-223 regulates proliferation and apoptosis by targeting insulin-like growth factor 1 receptor (IGF1R) in osteosarcoma cells. Mater. Express.

[32] Hilmi C., Larribere L., Giuliano S., Bille K., Ortonne J.P., Ballotti R., Bertolotto C. (2008). IGF1 Promotes Resistance to Apoptosis in Melanoma Cells through an Increased Expression of BCL2, BCL-X(L), and Survivin. J. Investig. Dermatol..

[33] Muraguchi T., Nanba D., Nishimura E.K., Tashiro T. (2019). IGF-1R deficiency in human keratinocytes disrupts epidermal homeostasis and stem cell maintenance. J. Dermatol. Sci..

[34] Bagordakis E., Sawazaki-Calone I., Macedo C.C.S., Carnielli C.M., De Oliveira C.E., Rodrigues P.C., Coletta R.D. (2016). Secretome profiling of oral squamous cell carcinoma-associated fibroblasts reveals organization and disassembly of extracellular matrix and collagen metabolic process signatures. Tumor Biol..

[35] Graizel D., Zlotogorski-Hurvitz A., Tsesis I., Rosen E., Kedem R., Vered M. (2020). Oral cancer-associated fibroblasts predict poor survival: Systematic review and meta-analysis. Oral Dis..

[36] Kellermann M.G., Sobral L.M., Silva S.D., Zecchin K.G., Graner E., Lopes M.A., Nishimoto I., Kowalski L.P., Coletta R.D. (2007). Myofibroblasts in the stroma of oral squamous cell carcinoma are associated with poor prognosis. Histopathology.

[37] Kashima H., Noma K., Ohara T., Kato T., Katsura Y., Komoto S., Fujiwara T. (2019). Cancer-associated fibroblasts (CAFs) promote the lymph node metastasis of esophageal squamous cell carcinoma. Int. J. Cancer.

[38] Buim M.E.C., Gurgel C.A.S., Gonçalves Ramos E.A., Lourenço S.V., Soares F.A. (2011). Activation of sonic hedgehog signaling in oral squamous cell carcinomas: A preliminary study. Hum. Pathol..

[39] De Faro Valverde L., De Almeida Pereira T., Dias R.B., Guimarães V.S.N., Ramos E.A.G., Santos J.N., Rocha C.A.G. (2016). Macrophages and endothelial cells orchestrate tumor-associated angiogenesis in oral cancer via hedgehog pathway activation. Tumor Biol..

[40] Fernandez C., Tatard V.M., Bertrand N., Dahmane N. (2010). Differential Modulation of Sonic-Hedgehog-Induced Cerebellar Granule Cell Precursor Proliferation by the IGF Signaling Network. Dev. Neurosci..

[41] Hsieh A., Ellsworth R., Hsieh D. (2011). Hedgehog/GLI1 regulates IGF dependent malignant behaviors in glioma stem cells. J. Cell. Physiol..

[42] Nakamura K., Sasajima J., Mizukami Y., Sugiyama Y., Yamazaki M., Fujii R., Kohgo Y. (2010). Hedgehog Promotes Neovascularization in Pancreatic Cancers by Regulating Ang-1 and IGF-1 Expression in Bone-Marrow Derived Pro-Angiogenic Cells. PLoS ONE.

[43] Lu Y., Li J., Cheng J., Lubahn D.B. (2015). Genes targeted by the Hedgehog-signaling pathway can be regulated by Estrogen related receptor β. BMC Mol. Biol..

[44] Zhou J., Zhu G., Huang J., Li L., Du Y., Gao Y., Wu K. (2016). Non-canonical GLI1/2 activation by PI3K/AKT signaling in renal cell carcinoma: A novel potential therapeutic target. Cancer Lett..

[45] Riobó N.A., Lu K., Ai X., Haines G.M., Emerson C.P. (2006). Phosphoinositide 3-kinase and Akt are essential for Sonic Hedgehog signaling. Proc. Natl. Acad. Sci. USA.

[46] Rao G., Pedone C.A., Valle L.D., Reiss K., Holland E.C., Fults D.W. (2004). Sonic hedgehog and insulin-like growth factor signaling synergize to induce medulloblastoma formation from nestin-expressing neural progenitors in mice. Oncogene.

[47] Singh R.K., Dhadve A., Sakpal A., De A., Ray P. (2016). An active IGF-1R-AKT signaling imparts functional heterogeneity in ovarian CSC population. Sci. Rep..

[48] Mohiuddin I.S., Wei S.J., Kang M.H. (2019). Role of OCT4 in cancer stem-like cells and chemotherapy resistance. Biochim. Biophys. Acta (BBA) Mol. Basis Dis..

[49] Lee J.J., Loh K., Yap Y.S. (2015). PI3K/Akt/mTOR inhibitors in breast cancer. Cancer Biol. Med..

[50] Singh S.K., Clarke I.D., Terasaki M., Bonn V.E., Hawkins C., Squire J., Dirks P.B. (2003). Identification of a cancer stem cell in human brain tumors. Cancer Res..

[51] Shah A., Patel S., Pathak J., Swain N., Kumar S. (2014). The Evolving Concepts of Cancer Stem Cells in Head and Neck Squamous Cell Carcinoma. Sci. World J..

[52] Gupta M.K., De Jesus D.F., Kahraman S., Valdez I.A., Shamsi F., Yi L., Kulkarni R.N. (2018). Insulin receptor-mediated signaling regulates pluripotency markers and lineage differentiation. Mol. Metab..

[53] Jacobo S.M.P., Kazlauskas A. (2015). Insulin-like Growth Factor 1 (IGF-1) Stabilizes Nascent Blood Vessels. J. Biol. Chem..

[54] Newman A.C., Nakatsu M.N., Chou W., Gershon P.D., Hughes C.C.W. (2011). The requirement for fibroblasts in angiogenesis: Fibroblast-derived matrix proteins are essential for endothelial cell lumen formation. Mol. Biol. Cell.

[55] Tai Y.T., Podar K., Catley L., Tseng Y.H., Shringarpure R., Burger R., Richardson P. (2003). Insulin-like growth factor-1 induces adhesion and migration in human multiple myeloma cells via activation of b1-integrin and phosphatidylinositol 3-kinase/AKT signaling beta. Blood.

[56] Shi X., Teng F. (2015). Down-regulated miR-28-5p in human hepatocellular carcinoma correlated with tumor proliferation and migration by targeting insulin-like growth factor-1 (IGF-1). Mol. Cell. Biochem..

[57] Murekatete B., Shokoohmand A., McGovern J., Mohanty L., Meinert C., Hollier B.G., Kashyap A.S. (2018). Targeting Insulin-Like Growth Factor-I and Extracellular Matrix Interactions in Melanoma Progression. Sci. Rep..

[58] Yao J., Yan M., Guan Z., Pan C., Xia L., Li C., Liu Q. (2009). Aurora-A down-regulates IkappaBα via Akt activation and interacts with insulin-like growth factor-1 induced phosphatidylinositol 3-kinase pathway for cancer cell survival. Mol. Cancer.

[59] Yoeli-Lerner M., Toker A. (2006). Akt/PKB signaling in cancer: A function in cell motility and invasion. Cell Cycle.

[60] Ma J., Sawai H., Matsuo Y., Ochi N., Yasuda A., Takahashi H., Takeyama H. (2010). IGF-1 Mediates PTEN Suppression and Enhances Cell Invasion and Proliferation via Activation of the IGF-1/PI3K/Akt Signaling Pathway in Pancreatic Cancer Cells. J. Surg. Res..

[61] Chung T.W., Lee Y.C., Kim C.H. (2004). Hepatitis B viral HBx induces matrix metalloproteinase-9 gene expression through activation of ERK and PI-3K/AKT pathways: Involvement of invasive potential. FASEB J..

[62] Nanta R., Shrivastava A., Sharma J., Shankar S., Srivastava R.K. (2018). Inhibition of sonic hedgehog and PI3K/Akt/mTOR pathways cooperate in suppressing survival, self-renewal and tumorigenic potential of glioblastoma-initiating cells. Mol. Cell. Biochem..

[63] Martins G.L.S., Paredes B.D., Sampaio G.L.A., Nonaka C.K.V., Da Silva K.N., Allahdadi K.J., Souza B.S.F. (2019). Generation of human iPS cell line CBTCi001-A from dermal fibroblasts obtained from a healthy donor. Stem Cell Res..

[64] Brady G., Crean S.J., Naik P., Kapas S. (2007). Upregulation of IGF-2 and IGF-1 receptor expression in oral cancer cell lines. Int. J. Oncol..

[65] Gonçalves G.V.M., Silva D.N., Carvalho R.H., Souza B.S.F., Da Silva K.N., Vasconcelos J.F., Soares M.B.P. (2017). Generation and characterization of transgenic mouse mesenchymal stem cell lines expressing hIGF-1 or hG-CSF. Cytotechnology.

[66] Doench J.G., Fusi N., Sullender M., Hegde M., Vaimberg E.W., Donovan K.F., Smith I., Tothova Z., Wilen C., Orchard R. (2016). Optimized sgRNA design to maximize activity and minimize off-target effects of CRISPR-Cas9. Nat. Biotechnol..

