# Supplementary material for: Effects of IGF-1 on Proliferation, Angiogenesis, Tumor Stem Cell Populations and Activation of AKT and Hedgehog Pathways in Oral Squamous Cell Carcinoma
Source: Int J Mol Sci. 2020 Sep 5;21(18):6487. doi: 10.3390/ijms21186487 (PMC7555130; doi:10.3390/ijms21186487)
Supplement: Supplementary file 1 [file ijms-21-06487-s001.pdf]

International Journal of
Molecular Sciences

Supplementary Materials

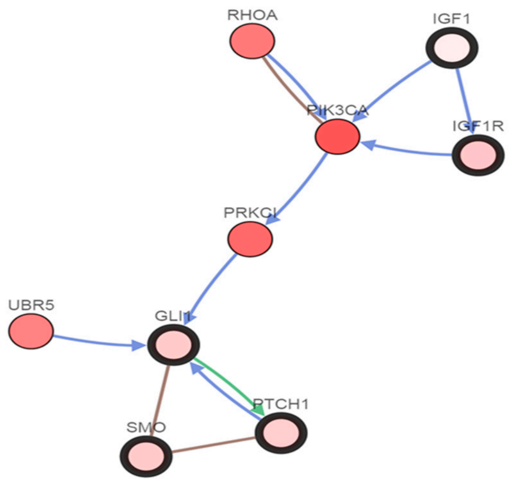

Figure S1. Illustration of communication network between intracellular signaling pathways. Circles represent different signaling pathways and arrows demonstrate the types and flow of communication accruing. Lilac arrows correspond to changes of state in the indicated direction. Green arrows indicate expression control in the corresponding pathways and state changes in the indicated pathways. As depicted in the figure, IGF-1 binding to the IGF1R receptor activates the PK3CA pathway, which, through PRKCI, promotes GLI1 expression. Sources: CERAMI et al., 2012; GAO et al., 2013.

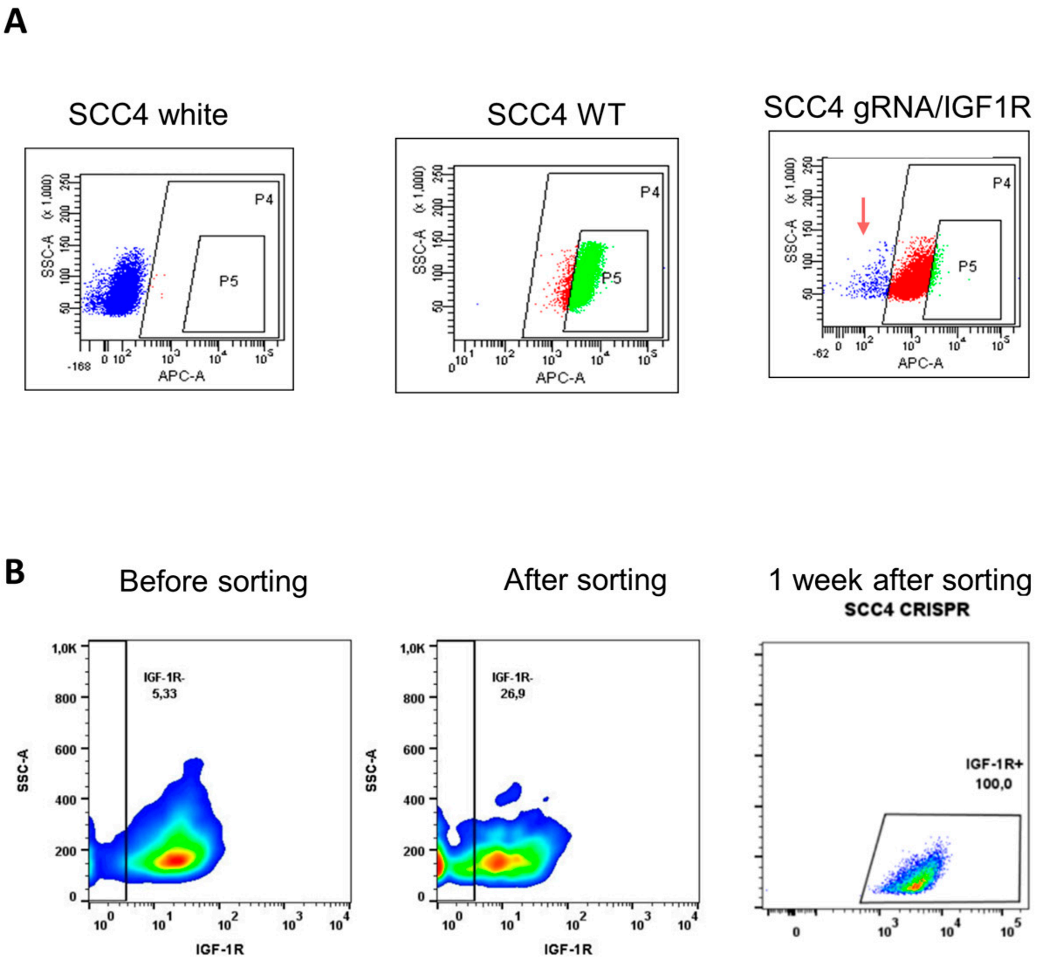

Figure S2. Flow cytometry gating data of SCC-4 CRISPR/IGF1R. The x-axis (APC-A/IGF1R) is the gate for fluorescent cells, while cell size is shown on the y-axis (SSC-A). (**A**) The first blue image represents the autofluorescence of SCC-4, as this sample was used as a control (white). The second image refers to anti-IGF1R antibody-labeled wild-type SCC-4 cells, in green, high SCC-4 receptor-labeled (gate P5) cells and cells with lower fluorescence signals indicating less labeling by the IGF1R antibody (gate P4). The last image refers to SCC-4 cells after receiving the CRISPR gRNA for IGF1R receptor deletion. After labeling the anti-IGF1R antibody, 3 subpopulations could be observed: gate P5 in green, cells with high IGF-1 expression (probably homozygous), gate P4 in red, intermediate expression (suggestive of heterozygous) and blue cells with low receptor expression (knockout). In order to select only knockout cells, cell-sorting was performed. (**B**) Before cell-sorting, the negative population for IGF1R represented 5.33%, as shown in the first graph. After cell-sorting, the population negative for IGF1R increased to 26.9%. However, one week after cell-sorting, when IGF1R expression was evaluated again, no IGF1R knockout cells were observed in culture (last image).

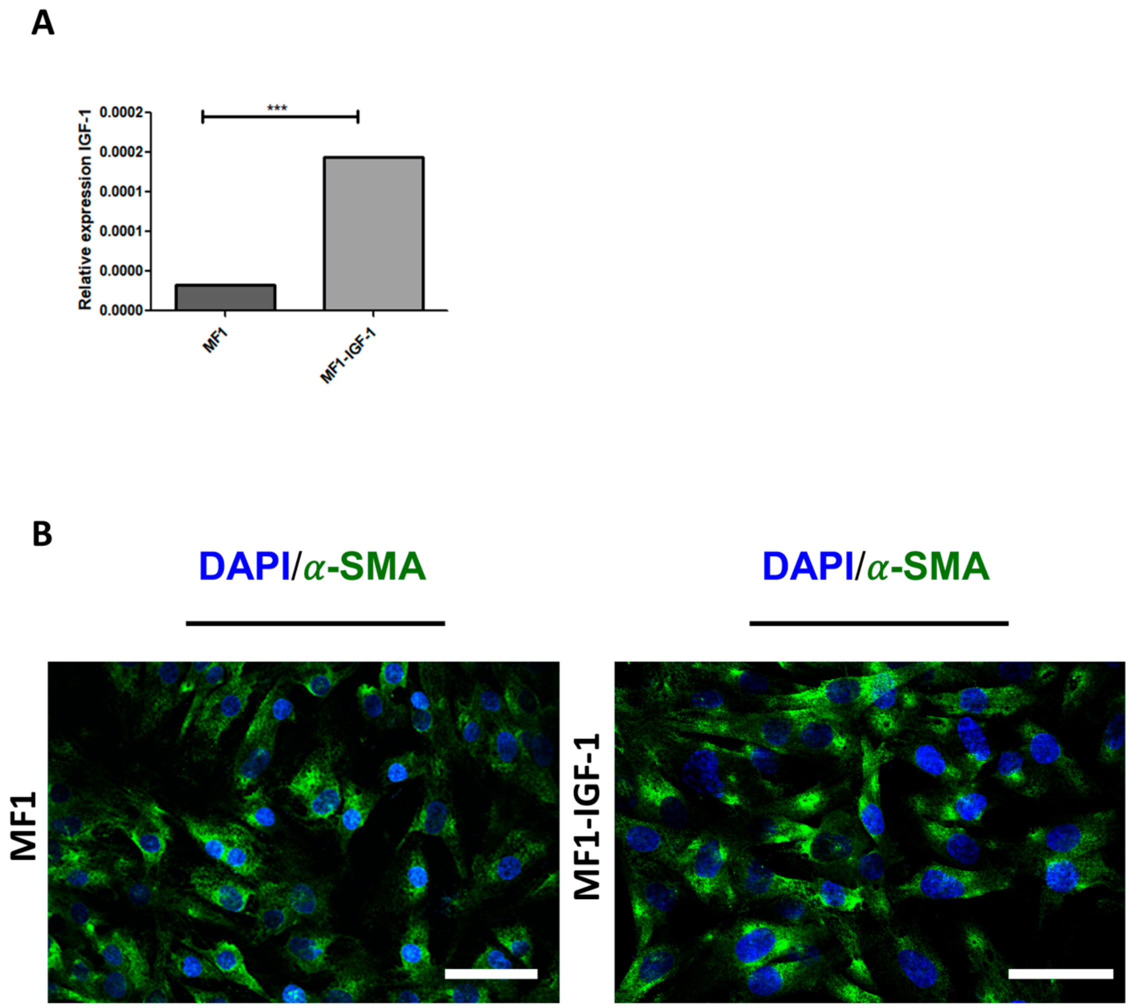

Figure S3. IGF-1 expression by qPCR and α-SMA immunostaining in MF1 and MF1-IGF-1 fibroblasts. (**A**) IGF-1 mRNA level in wild-type (MF1) and genetically modified (MF1-IGF-1) fibroblasts assessed by qPCR. A significant increase in IGF-1 expression can be observed in IGF-1-encoding lentivirus-modified fibroblasts. Bars represent comparisons between the respective groups and (***) denotes statistical significance after applying the one-way ANOVA and Dunnett's post-test, $p < 0.001$. (**B**) α-SMA immunostaining in MF1 and MF1-IGF-1 fibroblasts. High intensity α-SMA staining was observed in the cytoplasm in both cell lines (green). Nuclei were stained with DAPI (blue). Bars: 50 μm; 400× magnification.

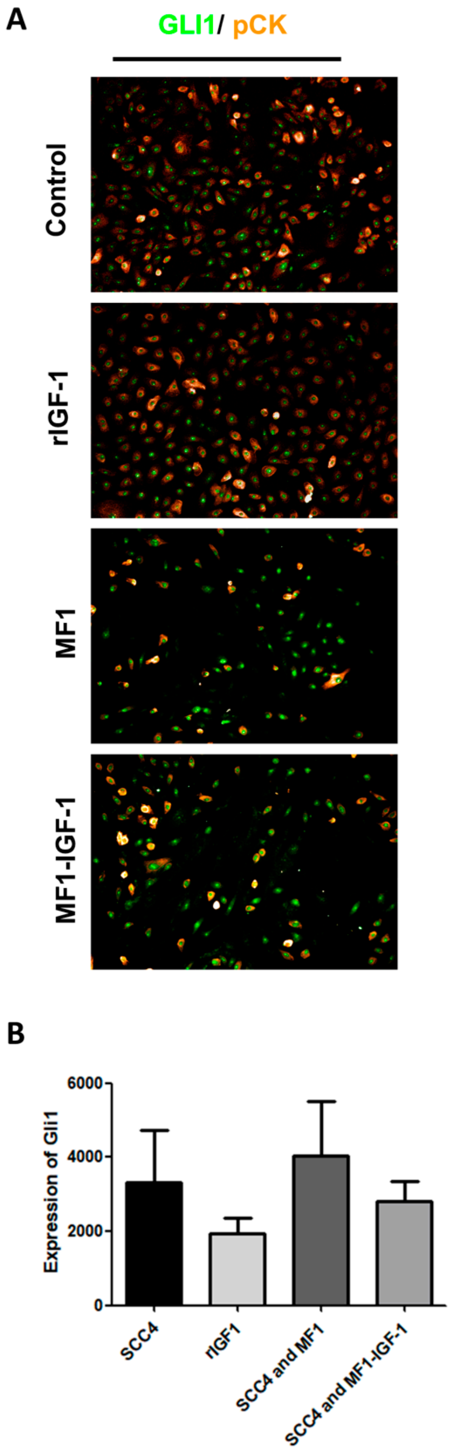

Figure S4. GLI1 expression in SCC-4 and fibroblast co-culture. (**A**) Cells were maintained in culture for 5 days, and then immunostained for GLI1 and cytokeratin. The following conditions were evaluated: control medium, rIGF-1, SCC-4 and MF1 or SCC-4 and MF1-IGF-1. GLI1 can be evidenced by positive green markings and orange cytokeratin (pCK). Images were obtained using the Operetta High Content Imaging System (Perkin Elmer, Waltham, MA, USA). Magnification: 100×. (**B**) Signal intensity quantification of GLI1 positivity in the nucleus and cytoplasm. The graph represents the expression levels of GLI1 under different stimuli. No significant differences in GLI1 expression were observed under the evaluated conditions.

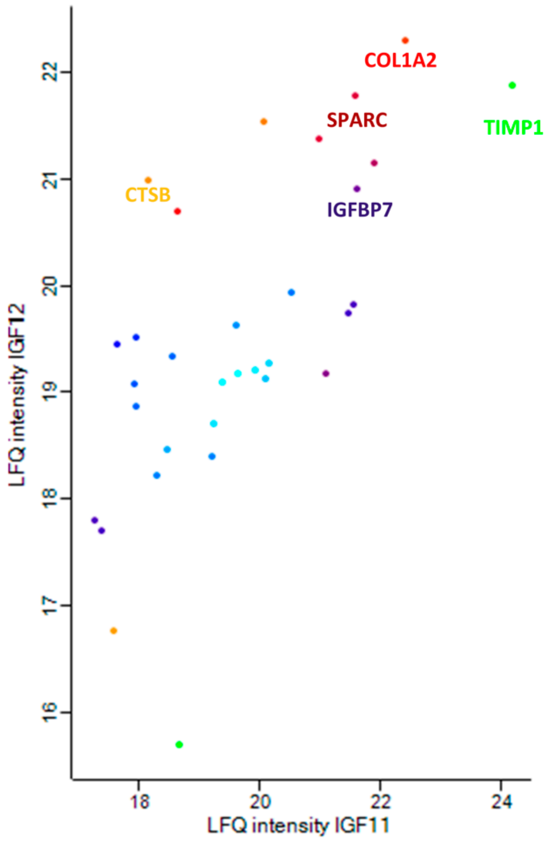

Figure S5. Principal component analysis (PCA). Distribution of proteins identified in MF1-IGF-1-conditioned medium according to intensity. Protein identification and quantification data were analyzed using the Perseus program, which considered the 24 most abundant peptides in MF1-IGF-1-conditioned medium in sample 1, and 22 of the most abundant peptides in MF1-IGF-1-conditioned medium in sample 2. The distribution of the identified proteins is represented by colored dots along the graph. Each point corresponds to a different protein. The most abundant proteins are shown at the top of the graph in relation to the *y*-axis and offset to the right in relation to the *x* axis. The main peptides identified in this analysis were COL1A2, TIMP1, SPARC, IGFBP7 and CTSB. COL1A2 = collagen alpha-2 (I) chain, TIMP1 = metallopeptidase inhibitor 1, SPARC = secreted protein acidic and cysteine rich, IGFBP7 = insulin like growth factor binding protein 7 and CTSB = cathepsin B.

© 2020 by the authors. Submitted for possible open access publication under the terms and conditions of the Creative Commons Attribution (CC BY) license (http://creativecommons.org/licenses/by/4.0/).
